# Restoration of ovarian function and natural fertility following the cryopreservation and autotransplantation of whole adult sheep ovaries

**DOI:** 10.1093/humrep/deu144

**Published:** 2014-06-17

**Authors:** B.K. Campbell, J. Hernandez-Medrano, V. Onions, C. Pincott-Allen, F. Aljaser, J. Fisher, A.S. McNeilly, R. Webb, H.M. Picton

**Affiliations:** 1 Division of Child Health Obstetrics and Gynaecology, School of Medicine, University of Nottingham, Nottingham NG7 2UH, UK; 2 School of Chemistry, University of Leeds, LeedsLS2 9JT, UK; 3 MRC Centre for Reproductive Health, University of Edinburgh, Edinburgh EH16 4TJ, UK; 4 Division of Animal Science, School of Biosciences, University of Nottingham, Nottingham LE12 5RD, UK; 5 Reproduction and Early Development, Leeds Institute of Genetics, Health and Therapeutics, University of Leeds, Leeds LS2 9JT, UK

**Keywords:** ovary, cryopreservation, fertility restoration, vascular pedicle

## Abstract

**STUDY QUESTION:**

Is it possible to restore ovarian function and natural fertility following the cryopreservation and autotransplantation of whole ovaries, complete with vascular pedicle, in adult females from a large monovulatory animal model species (i.e. sheep)?

**SUMMARY ANSWER:**

Full (100%) restoration of acute ovarian function and high rates of natural fertility (pregnancy rate 64%; live birth rate 29%), with multiple live births, were obtained following whole ovary cryopreservation and autotransplantation (WOCP&TP) of adult sheep ovaries utilizing optimized cryopreservation and post-operative anti-coagulant regimes.

**WHAT IS KNOWN ALREADY:**

Fertility preservation by WOCP&TP requires successful cryopreservation of both the ovary and its vascular supply. Previous work has indicated detrimental effects of WOCP&TP on the ovarian follicle population. Recent experiments suggest that these deleterious effects can be attributed to an acute loss of vascular patency due to clot formation induced by damage to ovarian arterial endothelial cells.

**STUDY DESIGN, SIZE, DURATION:**

Study 1 (2010–2011; *N* = 16) examined the effect of post-thaw perfusion of survival factors (angiogenic, antioxidant, anti-apoptotic; *n* = 7–8) and treatment with aspirin (pre-operative versus pre- and post-operative (*n* = 7–9)) on the restoration of ovarian function for 3 months after WOCP&TP. Study 2 (2011–2012; *N* = 16) examined the effect of cryoprotectant (CPA) perfusion time (10 versus 60 min; *n* = 16) and pre- and post-operative treatment with aspirin in combination with enoxaparine (Clexane^®^; *n* = 8) or eptifibatide (Integrilin^®^; *n* = 8) on ovarian function and fertility 11–23 months after WOCP&TP.

**PARTICIPANTS/MATERIALS, SETTING, METHODS:**

Both studies utilized mature, parous, Greyface ewes aged 3–6 years and weighing 50–75 kg. Restoration of ovarian function was monitored by bi-weekly blood sampling and display of behavioural oestrus. Blood samples were assayed for gonadotrophins, progesterone, anti-Müllerian Hormone and inhibin A. Fertility restoration in Study 2 was quantified by pregnancy rate after a 3 month fertile mating period and was confirmed by ultrasound, hormonal monitoring and live birth. Ovarian function was assessed at sacrifice by ovarian appearance and vascular patency (Doppler ultrasound) and by follicular histology.

**MAIN RESULTS AND THE ROLE OF CHANCE:**

In Study 1, survival factors were found to have no benefit, but the inclusion of pre-operative aspirin resulted in four ewes showing acute restoration of ovarian function within 3 weeks and a further six ewes showing partial restoration. The addition of post-operative aspirin alone had no clear benefit. In Study 2, combination of aspirin with additional post-operative anti-coagulants resulted in total acute restoration of ovarian function in 14/14 ewes within 3 weeks of WOCP&TP, with 9/14 ewes becoming pregnant and 4/14 giving birth to a total of seven normal lambs. There was no difference between anti-coagulants in terms of restoration of reproductive function and fertility. In contrast, the duration of CPA perfusion was highly significant with a 60 min perfusion resulting in ovaries of normal appearance and function with high rates of primordial follicle survival (70%) and an abundant blood supply, whereas ovaries perfused for 10 min had either resorbed completely and were vestigial (7/14) or were markedly smaller (*P* < 0.01). It is concluded that both the degree of CPA penetration and the maintenance of post-operative vascular patency are critical determinants of the success of WOCP&TP.

**LIMITATIONS, REASONS FOR CAUTION:**

Before application of this technology to fertility preservation patients, it will be critical to optimize the CPA perfusion time for different sized human ovaries, determine the optimum period and level of anti-coagulant therapy, and confirm the normality of offspring derived from this procedure.

**WIDER IMPLICATIONS OF THE FINDINGS:**

This technology holds promise for the preservation of fertility in women. It could also potentially be applied to the cryopreservation of other reproductive or even major organs (kidneys) where there are considerable difficulties in storing donated tissue.

**STUDY FUNDING/COMPETING INTEREST(S):**

Funding was received from the Medical Research Council, University of Nottingham. The authors confirm that they have no conflict of interest in relation to this work.

## Introduction

Premature ovarian failure (POF) affects 1–2% of women (Coulam *et al.*, 1986) and is due to a number of causes including genetic predisposition and gonadotoxic treatment (Aubard *et al.*, 2001; Kim *et al.*, 2001). This loss of ovarian function, in addition to primary infertility and amenorrhoea, can have far-reaching clinical and psychological effects such as failure to develop secondary sexual characteristics, loss of self-confidence, depression, and an increased risk of osteoporosis, and heart disease (Carr, 2003). The importance of POF and its impact on human health is increasing due to the increased incidence of childhood cancer (Byrne *et al.*, 1992) and paradoxically the continued improvement in cancer patient survival rates (Carr, 2003).

Ovarian tissue cryopreservation and autotransplantation (aTP) represents a potentially valuable technique in the preservation and restoration of fertility (Picton *et al.*, 2000). In instances when POF can be predicted, ovarian tissue, and the oocytes within it, can be removed and protected from damage or further deterioration in the frozen state and then returned to resume development when the patient requires the restoration of ovarian function (Campbell and Picton, 1999; Gosden *et al.*, 1999; Picton *et al.*, 2000). Following pioneering proof of principle studies which used sheep as a physiologically relevant model of ovarian function for the human (Gosden *et al.*, 1994; Baird *et al.*, 1999), restoration of fertility following cryopreservation and subsequent autografting of small pieces of ovarian cortical tissue has been used to preserve and restore the fertility of women, with reports of ∼25 live births following both spontaneous ovulation and natural conception and the use of interventions such as IVF (Donnez *et al.*, 2004, 2013; Demeestere *et al.*, 2007; Meirow *et al.*, 2007; Andersen *et al.*, 2008; Ernst *et al.*, 2010; Silber, 2012). While this success is encouraging, the transplanted fragments of ovarian cortex contain only a fraction of an individual's ovarian reserve and as such can only provide the recipient with a relatively brief fertile window before the supply of oocytes contained within their graft is depleted (Donnez *et al.*, 2004, 2013; Demeestere *et al.*, 2007; Meirow *et al.*, 2007; Andersen *et al.*, 2008; Ernst *et al.*, 2010; Silber, 2012). The problem of graft longevity is exacerbated by freeze-thaw damage and high rates of follicle loss due to ischaemia during revascularization of the transplanted tissue (Gosden *et al.*, 1994). These limitations mean that cryopreservation and autografting of pieces of cortex appears to be less effective as a means to restore the fertility of older patients in whom follicle density is already low at the time of tissue preservation and where it is associated with endocrine disturbance (Gosden *et al.*, 1994; Baird *et al.*, 1999; Campbell *et al.*, 2004). In marked contrast, cryopreservation of the whole ovary, complete with vascular pedicle, for later autotransplantation (WOCP&TP) provides an attractive alternative strategy for fertility preservation as it involves restoration of all of the primordial follicles within the ovary. Further, as aTP requires vascular anastomosis rather than cortical revascularization, this intervention should result in no marked diminution of ovarian reserve due to ischaemia, thus preventing endocrine imbalance and reducing the age constraints which limit the efficacy of this fertility preservation technology.

The concept of whole organ cryopreservation is attractive; however attempts to freeze-store vital organs such as the heart, liver and kidney have so far proved uniformly unsuccessful. The primary challenge with whole organ cryopreservation, as distinct from the preservation of cell suspensions or tissue fragments, is the crucial need to preserve an intact vascular system to allow for subsequent organ revascularization and function (Pegg, 2002, 2007). All methods of tissue cryopreservation require the replacement of intra-cellular water with penetrating cryoprotective agents (CPA) such as dimethyl sulphoxide (DMSO). During organ preservation this has usually been achieved by vascular perfusion. This technique has the advantage of providing very short diffusion distances for the CPA penetration, but conversely it has the potential to cause microvascular damage (Pegg, 1972, 2002, 2007). The mechanisms of cellular damage and the mode of action of CPAs are now well understood and it is clear that the cellular injury and loss caused by the formation of intra-cellular ice during cooling and warming can usually be avoided, whereas the formation of ice in extra-cellular locations, and particularly in blood vessels, can produce serious injury and prevent organ revascularization (Rubinsky and Pegg, 1988; Pegg, 2002, 2007).

Faced with these constraints of organ cryopreservation, to date, attempts to perform WOCP&TP in large animals have met with only limited success. Despite apparently high rates of post-thaw tissue viability following WOCP in both human (Bedaiwy *et al.*, 2006; Jadoul *et al.*, 2007; Martinez-Madrid and Donnez, 2007) and sheep (Onions *et al.*, 2008), aTP of these frozen ovaries in both lambs (Revel *et al.*, 2004; Arav *et al.*, 2005, 2010; Imhof *et al.*, 2006) and adult animals (Bedaiwy *et al.*, 2003; Onions *et al.*, 2008, 2009) have resulted in high rates of follicle loss and organ failure resulting in low rates of restoration of ovarian function and fertility. The report of a live birth from a lamb which had undergone WOCP&TP (Imhof *et al.*, 2006), albeit 545 days after aTP, and the fact that those animals that do show restoration of ovarian function continue to cycle for many years (Bedaiwy *et al.*, 2008; Arav *et al.*, 2010) does, however, illustrate the promise of this intervention for restoration of fertility. Recent experiments in our laboratory suggest that the deleterious effects on both the ovarian follicle population and surrounding ovarian tissue following WOCP&TP can be attributed, at least in part, to an acute loss of vascular patency that appears to be associated with significant changes in gene expression in arterial endothelial cells of local factors associated with vascular tone, wound repair and/or hypoxia (Onions *et al.*, 2013). In this paper, we report that optimization of CPA penetration during whole ovary perfusion and the use of anti-thrombotic agents to prevent post-operative clot formation in the ovarian vasculature results in a dramatic increase in the success rates of WOCP&TP in adult sheep, with the rapid restoration of ovarian function in all animals and good rates of natural fertility and multiple live births being achieved in adult animals.

## Materials and Methods

All materials were purchased from Sigma Aldrich, Dorset, UK, unless otherwise stated. All sutures were purchased from Ethicon, Edinburgh, UK.

### Whole ovarian cryopreservation and transplantation *in vivo*

#### Experimental animals

All animal procedures were carried out with approval from the UK Home Office and in accordance with the Animals (Scientific Procedures) Act 1986 following ethical review. Experimental animals were mature parous Greyface (cross between Scottish Blackface and Bluefaced/Border Leicester breeds) ewes 3–6 years old weighing 50–75 kg. Ewes were obtained from commercial sources and subject to veterinary inspection prior to use. Ewes were normally housed in group pens on deep straw bedding in a naturally ventilated and lit barn and fed a maintenance diet of ad libitum hay supplemented with ∼600 g/day concentrated sheep ration. At the time of surgery, general anaesthesia was induced using sodium thiopenthal (15 mg kg^−1^) with animals intubated and kept under inhaled anaesthesia with isofluorane (2.5–3%). Post-operative analgesia and antibiotic cover was provided in the form of a non-steroidal anti-inflammatory drug (2.2 mg kg^−1^; Finadyne, Shering Plough Animal Health, Herts, UK) and broad spectrum antibiotic (3–4 ml animal^−1^, Streptacare, Animal Care, Ltd, York, UK) for 3–5 days after surgery. Prior to aTP, each ewe was treated with a regime of progesterone and estradiol benzoate previously shown to increase ovarian and uterine blood flow (Brown and Mattner, 1984). This regime consisted of progestagen impregnated sponges (flugestone acetate; Chronogest CR, Intervet UK Ltd, Milton Keynes, UK) implanted intravaginally for 10–11 days, with sponge withdrawal and estradiol benzoate injection (100 µg animal^−1^) on the day before aTP.

#### Ovarian collection, perfusion and cryopreservation

Mid-ventral laparotomy was performed under general anaesthesia and the ovary and 10–15 cm of the ovarian vascular pedicle was carefully dissected, with side branch vessels being cauterized or tied off as appropriate, to a point just proximal of the bifurcation of the utero-ovarian vein into its ovarian and uterine branches. At this point, the vascular pedicle normally consists of just one uterine vein and a less tortuous ovarian artery and these vessels were ligated with a single suture (4/0 Mersilk), then excised and the ovary and pedicle removed. With the aid of an operating microscope (OPMI 6 SFR with Universal S2 stand microscope; Carl Zeiss mbH, Oberkochen, Germany), the ovarian artery was cannulated using a 2.5F (0.75 mm OD) intravenous cannulae (Sims Portex Limited, Kent, UK) in 2010 (Study 1) or a 22G Leaderflex cannula with extension (0.7 mm × 20 cm, Vygon GmbH & Co., Aachen, Germany) in 2011 (Study 2). The cannula was tied securely in place using two 0 mersilk (Ethicon, Edinburgh, UK) ligatures. The ovary and cannulated pedicle were then transferred to perfusion trays and subjected to cryoperfusion and cryopreservation, as detailed previously (Onions *et al.*, 2009). Briefly, the ovary was initially perfused, via the cannula, with cold, sterile, heparinised (100 IU ml^−1^) Ringer's solution for 10 min at a rate of 1 ml min^−1^ before being perfused with the cryopreservation media for either 10 (Study 2) or 60 min (Study 1 and 2) at a rate of 0.5 ml min^−1^ using a syringe driven perfusion pump (Precidor Infors Ag Basel; ChemLab Scientific Products Ltd, Hornchurch, UK) fitted with a 50 ml syringe. All solutions were refrigerated before use and all perfusions were carried out on ice. The cryopreservation media (CPA) was Leibovitz L-15 media supplemented with 1.5 mol l^−1^ DMSO, 0.1 mol l^−1^ sucrose and 10% (v/v) heat inactivated fetal calf serum (FCS). The media was adjusted to pH 7.3–7.4 and filter sterilized using a 0.22 nm syringe filter (Minisart^®^, Sartorius biotech, Surrey, UK) prior to use.

With the aid of a Planer controlled rate freezer (Kryo 550-16; Planer plc, Middlesex, UK) a slow-freezing cryopreservation protocol was utilized, which involved cooling to −9°C at −1°C min^−1^ before manual seeding was carried out. The temperature was then reduced at −0.2°C min^−1^ to −40°C following which the temperature was lowered at an increased rate of −10°C min^−1^ to a final temperature of −140°C. The cryogenic vials were then plunged into and stored in liquid nitrogen for at least 4 weeks prior to aTP.

#### Ovarian thawing

Each ovary was rapidly thawed in a 37°C water bath as described previously (Onions *et al.*, 2008) and transferred to a laminar flow hood. The ovary and pedicle were placed in a perfusion tray and immersed in and perfused with warm thawing media. Three thawing media were used, consisting of Leibovitz L-15 media supplemented with 10% FCS and reducing concentrations of DMSO (1 M, 0.5 M, 0 M). Sucrose was included only in the first thawing media at 0.1 M. All three thawing media (pH 7.3–7.4) were filter sterilized prior to use and perfused warm (37°C) through the ovary for 10 min at a rate of 1 ml min^−1^. The cannula was then eased from the ovarian artery ready for reanastomosis.

### Experimental designs

#### Study 1: effect of anti-thrombotic (aspirin) and survival factors on ovarian function following WOCP&TP

This initial WOCP&TP experiment, conducted over 2010–2011, examined the hypothesis that the deleterious effects of WOCP&TP on the ovarian follicle population and surrounding ovarian tissue could be attributed to an acute loss of vascular patency associated with clot formation in the ovarian arterial blood supply (Onions *et al.*, 2013). Two strategies were tested in order to minimize these vascular defects in a 2 × 2 factorial design utilizing 16 ewes in total: (i) the treatment of ewes with the anti-thrombotic aspirin (300 mg/day per os), either pre-operatively just prior to induction of anaesthesia for aTP (*n* = 9) or pre- and post-operatively for 5 days after surgery (*n* = 7); and (ii) the infusion of ‘survival factors’ (the anti-apoptotic factor: sphingosine-1-phosphate, the antioxidant: vitamin E, and the vascular factor: vascular endothelial growth factor) through the ovary and vascular pedicle post-thaw, prior to aTP (*n* = 7–8). Ewes were first randomized to survival factor treatment or not and then re-randomized to post-surgical aspirin treatment or not so that experimental groups were balanced according to number, animal age and weight. The slight imbalance in the final design was due to one ewe not being operated on due to severe mastitis and a technical error resulting in one ewe not receiving post-operative aspirin.

In this study, only one ovary was removed from each ewe for cryopreservation, as described above, at the time of initial laparotomy, with the contralateral ovary being left undisturbed for later collection to act as a time zero control. Either the left or right ovary was removed for cryopreservation on a random basis so groups were balanced. At least 4 weeks after cryopreservation, frozen ovaries were thawed as described above and ewes were randomized to either infusion with the final equilibration wash of Leibovitz L-15 media with 10% FBS (TM3) alone (*n* = 8) or TM3 further supplemented with vascular endothelial growth factor (VEGF 121; 10 ng/ml), sphingosine-1-phosphate (S-1-P; 170 µM) and Vitamin E (0.6 mM; *n* = 7) for one hour prior to transplantation.

All ewes received 300 mg of dispersible aspirin dissolved in water given orally immediately prior to induction of general anaesthesia for autotransplant surgery. During transplant re-equilibration, a transplantation site was prepared in the appropriate recipient ewe following a second laparotomy under general anaesthesia. This was accomplished by dissection of the ovarian vein and artery from the contralateral side so that freshly excised patent vessels were available for end-to-end micro-anastomosis to the ovarian artery and vein of the cryopreserved autotransplant, utilizing 10/0 and 8/0 Ethilon sutures respectively. The surgery was performed by a highly experienced vascular surgeon (B.K.C.) with the aid of a Zeiss operating microscope. The fresh ovarian and vascular tissue from the contralateral ovary was placed in Bouin's fixative for later histological analysis. All ewes received 5000 IU of heparin (i.v.) immediately prior to removal of vascular clamps to allow re-perfusion of blood through the transplant. Adequate re-perfusion was achieved with all autotransplants and the autotransplanted ovary and pedicle were attached to the uterine horn in as natural a position as possible with 6/0 Prolene sutures. After careful checking that haemostasis had been achieved, the abdominal wall was closed and the animal was allowed to recover, with 7 of the animals receiving a further 300 mg of post-operative aspirin daily for 7 days after the surgery. Commonly heat lamps were used during the recovery period and the animals were observed closely for signs of post-operative haemorrhage. The animals were kept in individual pens for around 7 days after the second surgery and were then moved to group pens for endocrine and behavioural monitoring. This consisted of collection of samples of jugular venous blood at 3–4 days intervals for 3 months and the detection of behavioural oestrus by inclusion of 1–2 vasectomized rams within the flock that were fitted with a marking device to record mating behaviour. At the end of this 3 month period, all ewes were sacrificed for tissue collection.

#### Study 2: the effect of time of CPA perfusion and post-operative anti-thrombotic therapy on restoration of ovarian function and fertility following WOCP&TP

**Figure 1:**
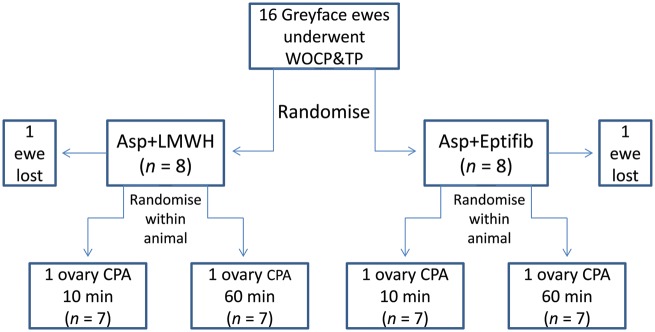
Illustration of the experimental design utilized for Study 2 which was conducted over the breeding season of 2011–2012. This experiment utilized 16 Greyface ewes who all underwent WOCP&TP. In addition to pre-operative Aspirin treatment and the intravenous injection of heparin prior to autotransplant revascularization, these ewes were randomized to two post-operative anti-coagulant regimes (*n* = 8) which involved the combination of aspirin treatment with either the injectable low molecular weight heparin enoxaparin (Clexane^®^; Asp+LMWH) or the glycoprotein IIb/IIIa inhibitor eptifibatide (Integrilin^®^; Asp+Eptifib). To further test the relative effectiveness of infusion of CPA for either 10 or 60 min prior to cryopreservation, the sheep were re-randomized within animal to allocate either the left or right ovary of each ewe to either the 10 or 60 min perfusion groups. These groups were balanced and great care was taken during the double autotransplantation procedure, which was performed at the same time, that there was no bias due to differences in the post-thaw period prior to re-anastomosis, surgeon fatigue or level of anti-coagulants in the circulation at the time of autotransplant revascularization. The actual experimental group sizes for analysis were reduced to *n* = 7 due to one animal not receiving pre-operative aspirin treatment and one animal not being able to receive a double ovarian autotransplant due to damage to the uterine artery during initial surgery. Statistical analysis of this design was simplified by the fact that both anti-coagulant regimes proved equally effective in restoring ovarian function and fertility and these data were therefore pooled to allow a paired analysis within animal of the effect of CPA perfusion time with *n* = 14.

On the basis of results obtained from Study 1 and subsequent *in vitro* studies, this final experiment was conducted over 2011–2012 and tested whether either a 10 or 60 min CPA perfusion supported superior restoration of ovarian function following WOCP&TP. This experiment involved a direct within-animal comparison with one ovary in each ewe being perfused with CPA for 10 min and the other being perfused for 60 min prior to WOCP (see Fig. 1). Similarly, on the basis that vascular CPA toxicity would not be limited by the shorter perfusion times, the effect of an extended post-operative regime of anti-thrombotic agents was tested in order to reduce post-transplant ischaemia by preventing clot formation in the ovarian vasculature induced by cryo- and cyto-toxic damage to the arterial endothelial cells (Onions *et al.*, 2013). The two regimes tested, combined pre- and post-operative aspirin treatment with either the injectable low molecular weight heparin enoxaparin (Clexane^®^; Asp+LMWH(Hep)) or the glycoprotein IIb/IIIa inhibitor eptifibatide (Integrilin^®^; Asp+Eptifib(Integ)). Due to concerns over the ability of sheep to tolerate these drugs, the doses used were relatively low and were well tolerated by the animals with no obvious adverse effects and 100% post-operative survival.

This experiment utilized 16 ewes and these animals were randomized according to animal number, age and weight to either of the post-operative anti-coagulant regimes (*n* = 8) and then re-randomized within animals to allocation of left or right ovaries to either 10 or 60 min of CPA perfusion so groups were balanced (*n* = 8; see Fig. 1). However, actual experimental group sizes were reduced to *n* = 7 due to one animal not receiving pre-operative aspirin treatment and one animal not being able to receive a double ovarian autotransplant due to damage to the uterine artery during initial surgery (Fig. 1).

Thus in Study 2 (2011–2012), both ovaries were removed for cryopreservation as described above, at initial laparotomy. The first ovary removed was perfused with 30 ml of CPA over a 60 min period and the second ovary removed was perfused with 5 ml of CPA over a 10 min period, before both ovaries were cryopreserved in the same run of a Planer controlled rate freezer as described above.

Both ovaries were returned in a single operation a minimum of one month after cryopreservation. As in Study 1, in the 10–11 days prior to aTP, all animals received a pretreatment with progesterone and estradiol benzoate in order to boost uterine blood flow (see above) and prior to induction of general anaesthesia all ewes received 300 mg of dispersible aspirin dissolved in water and given orally. The thawing of each ovary to be autotransplanted, as described above, was carefully timed so that the ovary was taken from liquid nitrogen 60–70 min prior to the estimated time of re-anastomosis. Each ovary was returned to its original position utilizing an end-to-side micro-anastomosis of the ovarian artery to the appropriate uterine artery (10/0 Ethilon) and the ovarian vein to an appropriately sized uterine vein (8/0 Ethilon) by the same experienced surgeon (B.K.C.). The autotransplanted ovary and pedicle were attached to the appropriate uterine horn in as natural a position as possible with 6/0 Prolene sutures. As in Study 1, all ewes received 5000 IU of heparin (i.v.) immediately prior to removal of vascular clamps to allow re-perfusion of blood through the first ovarian autotransplant. To make sure there was no experimental bias in terms of thawing times, surgeon fatigue and circulating heparin levels, the left ovary was thawed and autotransplanted first and the right ovary thawed and autotransplanted second. In this way an equal number of ovaries perfused with CPA for 10 and 60 min were autotransplanted first. Re-vascularization of the autotransplant was achieved in all instances and there were no post-operative complications.

In this study, as detailed above, the same regime of pre-operative and operative aspirin and heparin treatment was applied as was utilized in Study 1, but in addition post-operatively the animals received 300 mg of aspirin (per os) in combination with either 20 mg injectable heparin (Enoxaparin sodium 2000 units s.c.; Clexane^®^ Rhone-Poulenc Rorer; *n* = 8) or eptifibatide (2 mg ml^−1^ i.v.; Integrilin^®^ Glaxo-Smith Kline; *n* = 8) daily for 7 days after aTP. The eptifibatide was initially given at a dose of 10 mg/day for the initial 48 h after surgery and then 2 mg/day for the following 5 days. Due to concerns over the ability of sheep to tolerate these drugs after major surgery, these doses of enoxaparin (LMWH: 50%) and eptifibatide (10%) were significantly lower than those recommended by the manufacturer for use in humans for treatment of myocardial infarction (BNF, 2008).

From the time of aTP, all animals had samples of jugular venous blood collected at 3–4 days intervals and in addition for detection of behavioural oestrus ewes were run in a group with 1–2 entire rams which were fitted with a marking device to record mating behaviour. These ewes were also subjected to trans-abdominal ultrasound scanning for pregnancy detection at ∼15, 17 and 20 weeks post-aTP.

### Tissue collection

Under terminal general anaesthesia, the reproductive tracts were visualized following mid-ventral laparotomy and the size and appearance of the ovary(s), uterus and vascular pedicle were noted and photographed. The presence of adhesions was also recorded. Conventional and Power Doppler ultrasound (15–7io transducer, iU22 xMATRIX Ultrasound System, Royal Phillips Electronics, Amsterdam, The Netherlands) was used to confirm the presence or absence of antral follicles and blood flow through the ovary and these images were stored for later analysis. The animals were then sacrificed by an overdose of sodium pentobarbitone and the ovary complete with pedicle (if present) was fixed for later histological analysis. In Study 2, animals which had become pregnant and carried lambs to term were not sacrificed until October 2013, 22 months after aTP.

### Histological analyses

The fixed tissue samples were processed using an ascending series of alcohols and cleared using xylene. The tissue samples were then paraffin wax embedded for subsequent histological evaluation to quantify the follicle population as previously described (Onions *et al.*, 2013). Follicle count data were expressed per unit volume of ovarian tissue and subject to square root transformation prior to analysis by one way ANOVA or paired and unpaired *t*-test as was appropriate.

### Hormone analysis

Blood samples were centrifuged at 4°C and 3000*g* for 15 min to obtain plasma which was stored at −20°C until assay. FSH (Onions *et al.*, 2009), LH (Onions *et al.*, 2009) and progesterone (Campbell *et al.*, 1994) were determined by radioimmunoassay as previously described apart from the progesterone assay which utilized a different antibody (SAPU R7044X (Campbell *et al.*, 1998)). All assays had intra-assay and inter-assay coefficients of variation of <10 and 15% respectively. In Study 1, hormone profiles between individuals were too variable to meaningfully average but in Study 2, it was possible to generate and analyse mean profiles, particularly over the breeding season. The effect of post-operative type of anti-coagulant therapy on hormone profile data was therefore analysed using repeated measures ANOVA, with data being log transformed prior to statistical analysis.

Anti-Müllerian hormone (AMH) levels were determined by a competitive enzyme immunoassay kit (CSB-E12756h; Cusabio Biotech Co., Ltd, Hubei, China) adapted for ovine samples. Briefly, 50 µl of either sample or standards were added to corresponding wells, followed by 50 µl of horseradish peroxidase conjugate and 50 µl of anti-AMH antibody. After 60 min incubation at 37°C, plates were washed three times, then substrate was added (100 µl) and incubated in the dark for 15 min at 37°C. The reaction was stopped and visualized at a wavelength of 450 nm (with correction at 630 nm; Benchmark, Bio-Rad Laboratories, Hercules, CA, USA). AMH concentrations were calculated from the corresponding standard curve using Microplate Manager^®^ v5.2.1 software (Bio-Rad Laboratories). The assay sensitivity was 0.38 ng ml^−1^ with inter- and intra-assay coefficients of variation of <15%, respectively.

Inihibin A circulatory concentrations were measured using a modified two-site immunoassay (ELISA) as previously reported (McNeilly *et al.*, 2002), using an inhibin βA subunit monoclonal antibody as the capture antibody (Groome *et al.*, 1990) and an αC-specific biotinylated monoclonal antibody (Bleach *et al.*, 2001). Assay sensitivity was 32 pg ml^−1^, with 14.5 and 7.5% of inter- and intra-assay %CV, respectively.

### Determinations of CPA penetration and toxicity *in vitro*

CPA penetration and toxicity were determined by nuclear magnetic resonance (NMR) and lactate dehydrogenase (LDH) assays.

Sheep reproductive tracts were obtained from a local abattoir and the ovaries, complete with vascular pedicle, were dissected and the ovarian arteries cannulated as described above. Following cannulation, the ovary was then flushed with cold (0–4°C) heparinized Ringer's solution to remove blood and to confirm correct cannula placement. Ovaries were randomly assigned to treatment groups consisting of different periods of exposure during CPA infusion; 0 (control), 3, 10, 15, 30 and 60 min of perfusion. The rate of CPA infusion utilizing a syringe driven pump was 0.5 ml/min.

To quantify the relationship between CPA penetration and perfusion time, duplicate samples of ovarian cortex, medulla and vascular pedicle were collected with the aid of a biopsy punch (3 mm). Surface moisture was removed from each biopsy using filter paper and tissue was immersed in 600 µl of Deuterium oxide (D_2_O, 99.9%; Sigma, London, UK) in 1.7 ml Eppendorf tubes with a cap. The tube was then covered with parafilm and the cap secured. The tubes were frozen and stored at −20°C until the time of NMR analysis as previously described (Newton *et al.*, 1998). At least three replicate runs with two replicates per treatment per run were conducted for each determination (*n* = 6).

To quantify the relationship between CPA perfusion time and tissue toxicity, following perfusion the ovaries and vascular pedicle were subjected to slow freezing and thawing as described above. Tissue biopsies of ovarian cortex, medulla and pedicle (*n* = 6) were transferred into 24-well culture dish containing 1 ml of warm (37°C) culture media (M199 supplemented with HEPES, antibiotics and anti-mycotics) and cultured overnight at 37°C with 5% CO_2_. The culture media was then aspirated and assayed for LDH in triplicate (Cytotoxicity Detection Kit, Roche 11644793001; Burgess Hill, UK). LDH data were log transformed and analysed by ANOVA with Tukey's *post hoc* test.

## Results

### Study 1 (2010–2011): effect of anti-thrombotic (aspirin) and survival factors on ovarian function following WOCP&TP

In comparison to previous WOCP&TP experiments in our laboratory (Onions *et al.*, 2009, 2013) in which 5000 IU heparin was routinely administered just prior to transplant re-vascularization, the inclusion of 300 mg of pre-operative aspirin in the anti-coagulant regime given to all ewes had an unexpected, but markedly beneficial effect on the restoration of ovarian function following WOCP&TP. Of the 15 ewes, 10 showed evidence of at least partial restoration of ovarian function (Table I).

**Table I:** Summary of results from WOCP+TP study conducted in 2010–2011 (Study 1) testing the effect of exposure of the frozen ovary to survival factors post-thaw and the inclusion of aspirin in the anti-coagulation regime.

Characteristic	No restoration	Partial restoration	Full restoration
Number animals	5	6	4
Progesterone >0.3 ng/ml	No	Yes	Yes
Display oestrus	No	No	Yes
Mean FSH (ng/ml ± SEM)	11.8 ± 0.2^a^	10.2 ± 1.0^a^	5.4 ± 1.3^b^
Mean LH (ng/ml ± SEM)	2.6 ± 0.2^a^	2.5 ± 0.2^a^	1.4 ±0. 2^b^
Mean AMH (ng/ml ± SEM)	4.8 ± 1.7^a^	21.9 ± 6.9^b^	26.0 ± 7.4^b^
Ovarian tissue section area (Mean ± SEM; mm^2^)	47.0 ± 14.7^a^	62.1 ± 15.3^a^	172.1 ± 0.24^b^
Pretreatment (time 0) primordial and pre-antral follicle density (Follicles/mm^3^)*	3.09 ± 0.57^a^	3.68 ± 0.84^a^	2.48 ± 0.98^a^
Post-treatment primordial and pre-antral follicle density (follicles/mm^3^)*	0.64 ± 0.21^a^	0.70 ± 0.27^a^	1.43 ± 0.10^b^

Aspirin was administered either pre-operatively only or both pre-operatively and post-operatively. Data have been analysed on the basis of sheep which showed no, partial or full restoration of ovarian function within 3 months of autotransplantation of cryopreserved ovaries. Different superscripts indicate significant differences within rows (*P* < 0.05).

*Values subject to square root (+0.5) transformation prior to analysis.

**Figure 2:**
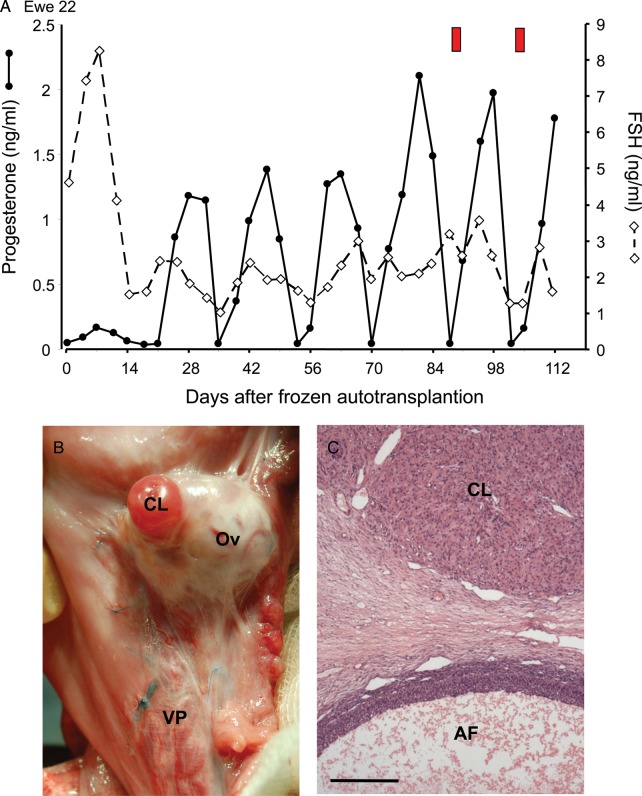
Results from the 2010–2011 WOCP&TP study (Study 1). (**A**) Hormone profiles from a single ewe (Ewe 22) who showed full restoration of ovarian function following WOCP&TP. Note the rapid decline in circulating FSH to normal levels within 14 days of autotransplantation, with ovulation occurring after 21 days, as evidenced by the initiation of a series of cyclic fluctuations in progesterone indicative of regular estrous cycles. Red bars indicate times at which oestrus activity was observed in this ewe. (**B**) Histological appearance of cryopreserved ovary (Ov) with an early cycle corpus luteum (CL) and morphologically normal vascular pedicle (VP) 3 months after autotransplantation. The blue 5/0 Prolene sutures used to attach the ovarian pedicle to the uterine wall can be clearly seen. (**C**) Histological section through an ovary shown in (B) illustrating normal appearance and vascularity of ovarian structures with a normal corpus luteum (CL) and a healthy large antral follicle (AF) evident in the section. Scale bar 500 µm.

Four of these animals had full ovarian function returned within 4–7 (5.4 ± 0.9 mean ± SEM) weeks of aTP (Fig. 2) and all showed regular oestrous cycles which were characterized by cyclic fluctuations in progesterone, the display of oestrus behaviour at normal intervals (15–20 days) and a rapid decline in FSH and LH concentrations to normal levels following aTP (Fig. 2A: LH not shown). The ovaries of these four animals were of normal appearance and vascularity (Fig. 2B) and contained morphologically normal well vascularized corpora lutea and antral follicles (Fig. 2B and C). However, the population of primordial and pre-antral follicles in the WOCP&TP animals which resumed oestrous cycles was estimated to be only around 20% of the normal ovarian population present in untreated control animals (Table I).

The five animals which showed no evidence of restoration of ovarian function following WOCP&TP had constantly high levels of FSH (>10 ng/ml) and low levels of progesterone (<0.2 ng/ml) and their ovaries and vascular pedicle had resorbed and were vestigial at the end of the 3 month monitoring period (Table I).

The remaining six animals, which showed a partial restoration of ovarian function, exhibited highly variable fluctuations in FSH (Table I) and progesterone levels (not shown) across the monitoring period and the ovaries were markedly smaller (*P* < 0.01; Table I) with significantly fewer (*P* < 0.05) primordial and pre-antral follicles (Table I) than normally cycling animals. As expected, the circulating levels of the ovarian reserve marker, AMH, were found to be very low in animals with no restoration of ovarian function, but did not differ between animals with partial and full restoration of ovarian cyclicity (Table I).

Contrary to expectations, however, neither inclusion of survival factors in the final re-equilibration perfusate used immediately prior to aTP or continuation of aspirin treatment alone for 5 days after aTP showed any evidence of having a beneficial effect on the rate of restoration of fertility following WOCP&TP. Thus, of the four animals which displayed full acute restoration of ovarian function, only two had received survival factors and only two had received post-operative aspirin. Similarly, of the six ewes which showed partial restoration of ovarian function, only three had received survival factors and only two had received post-operative aspirin. Examination of the possible effect of different initial primordial follicle density derived from Time 0 controls on subsequent restoration of ovarian function also showed no apparent relationship, as the ewes which showed full restoration had the lowest overall follicle counts (Table I). Overall, the results of Study 1 represented a significant advance in the success of WOCP&TP by demonstrating that the incorporation of a pre-operative treatment with anti-thrombotic aspirin was able to restore full ovarian function in around a quarter of the animals that underwent WOCP&TP within 4–7 weeks of surgery. However, the rate of restoration of ovarian function (25%) and the relatively low rate of primordial follicle survival in cycling animals (29%) were still far below the levels needed for this technique to be adopted as a clinical intervention. An additional series of *in vitro* experiments were therefore undertaken to further optimize the WOCP protocol and assess the possibility that CPA toxicity may explain the relatively high rate of loss of ovarian follicles following WOCP&TP.

### Determination of the effect of time of CPA infusion on CPA penetration and toxicity

**Figure 3:**
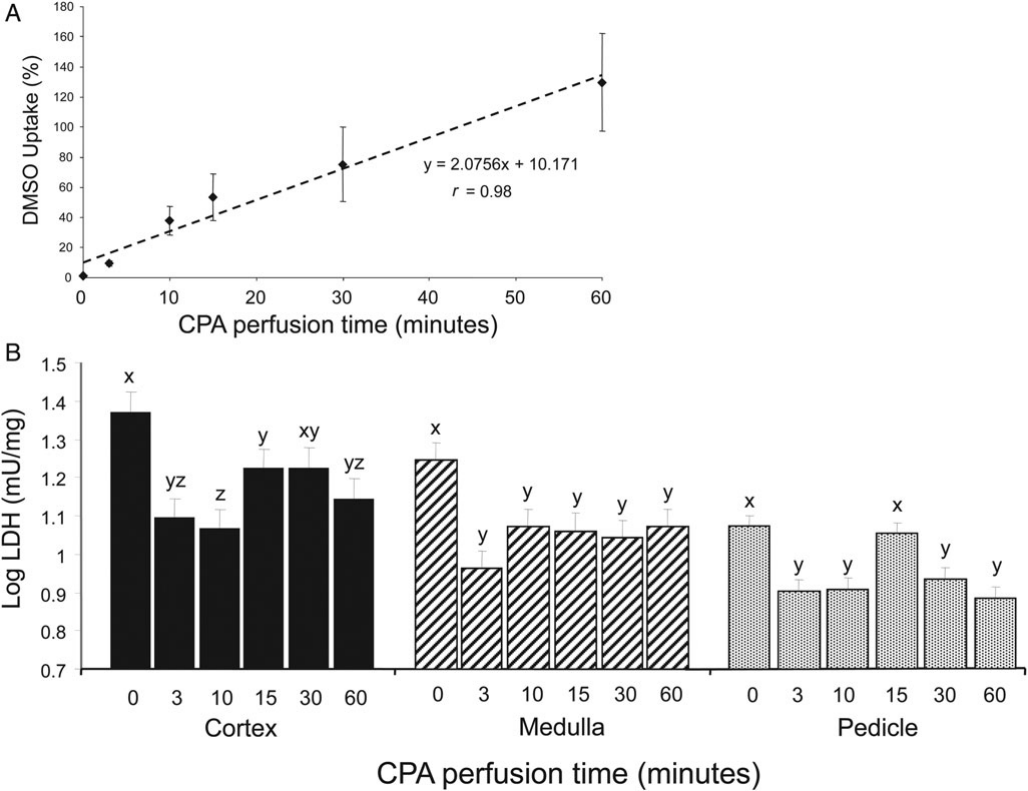
Graphs showing CPA penetration in the ovarian cortex (**A**) and the effect of CPA perfusion time (**B**) on the production of LDH (as an index of tissue damage) by ovarian cortex, medulla and pedicle. Values are mean ± SEM. Different letters indicate statistically significant differences at *P* < 0.05.

The primary challenge of organ cryopreservation is the necessity to maintain the functionality of multiple cell types within the organ whilst maintaining the integrity of the vascular supply post freeze-thaw. To address this question, we initially determined the relationship between the period of CPA perfusion of the ovarian pedicle and the degree of CPA penetration into the ovarian cortex, in which the primordial follicles are located. These data, determined by NMR spectrophoretic analysis (Fig. 3A), revealed a linear relationship (*r* = 0.98; *P* < 0.001) between 0–60 min of perfusion with CPA uptake of ∼100% after 60 min. We therefore set out to determine whether shorter periods of CPA exposure could limit damage to the ovarian vascular tissue due to CPA toxicity whilst maintaining penetration and cryoprotection of the ovarian cortex. Release of the enzyme LDH during overnight incubation of frozen/thawed tissue isolated from the ovarian cortex, medulla and vascular pedicle was used as an index of cryodamage and CPA toxicity. The data indicate that WOCP without CPA (0 perfusion time) resulted in the highest level of LDH release for all tissues, thus reflecting substantial cryodamage (Fig. 3). There were, however, marked differences in the relationship between LDH release and period of CPA exposure between the ovarian cortex and the vascular ovarian medulla and pedicle (Fig. 3b; *P* < 0.001).

Within the ovarian cortex, the lowest level of LDH release, and therefore cellular damage, was detected following the shortest periods of CPA perfusion (3 and 10 min), with higher levels at 15 and 30 min (*P* < 0.05) and with 60 min (our standard perfusion time) being intermediate between these two extremes. This result suggested that the 60 min period of CPA perfusion that had been used during WOCP&TP may have been resulting in CPA-induced cytotoxic damage to the ovarian cortex. This hypothesis was supported by the NMR analysis which showed very high rates of CPA uptake by the ovarian cortex at this infusion period (Fig. 3A). In contrast to the ovarian cortex, LDH release by the more vascular tissue of the ovarian medulla and pedicle did not differ significantly between CPA perfusion times of 3–60 min, apart from an increase at 15 min perfusion in the ovarian pedicle (Fig. 3B). This suggested that in vascular tissue, cryodamage due to lack of CPA exposure may be balanced by CPA toxicity resulting from the rapidity of CPA penetration. It therefore appeared unlikely that we would be able limit CPA toxicity to vascular tissue by reducing perfusion times.

Overall, the results of these *in vitro* optimization studies demonstrated that although the 60 min perfusion time afforded sufficient cryoprotection to both the ovarian cortex and the cortex-medullar interface to maintain the small antral follicle population following WOCP, shorter periods of CPA perfusion may be advantageous in terms of limiting potential CPA toxicity in the cortex whilst maintaining adequate cryoprotective cover in the ovarian vasculature.

### Study 2 (2011–2012): the effect of time of CPA perfusion and post-operative anti-thrombotic therapy on the success of WOCP&TP *in vivo*

On the basis of results obtained both Study 1 and from the *in vitro* studies described above, a second study, which was conducted over 2011–2012, tested whether a 10 or 60 min CPA perfusion supported superior restoration of ovarian function following WOCP&TP and whether an extended post-operative regime of anti-thrombotic agents would reduce post-transplant ischaemia by preventing clot formation in the ovarian vasculature induced by cryo- and cyto-toxic damage to the arterial endothelial cells (Onions *et al.*, 2013). The two regimes tested, as stated above, combined pre- and post-operative aspirin treatment with either the injectable low molecular weight heparin enoxaparin (Asp+LMWH) or the glycoprotein IIb/IIIa inhibitor eptifibatide (Asp+Eptifib).

**Figure 4:**
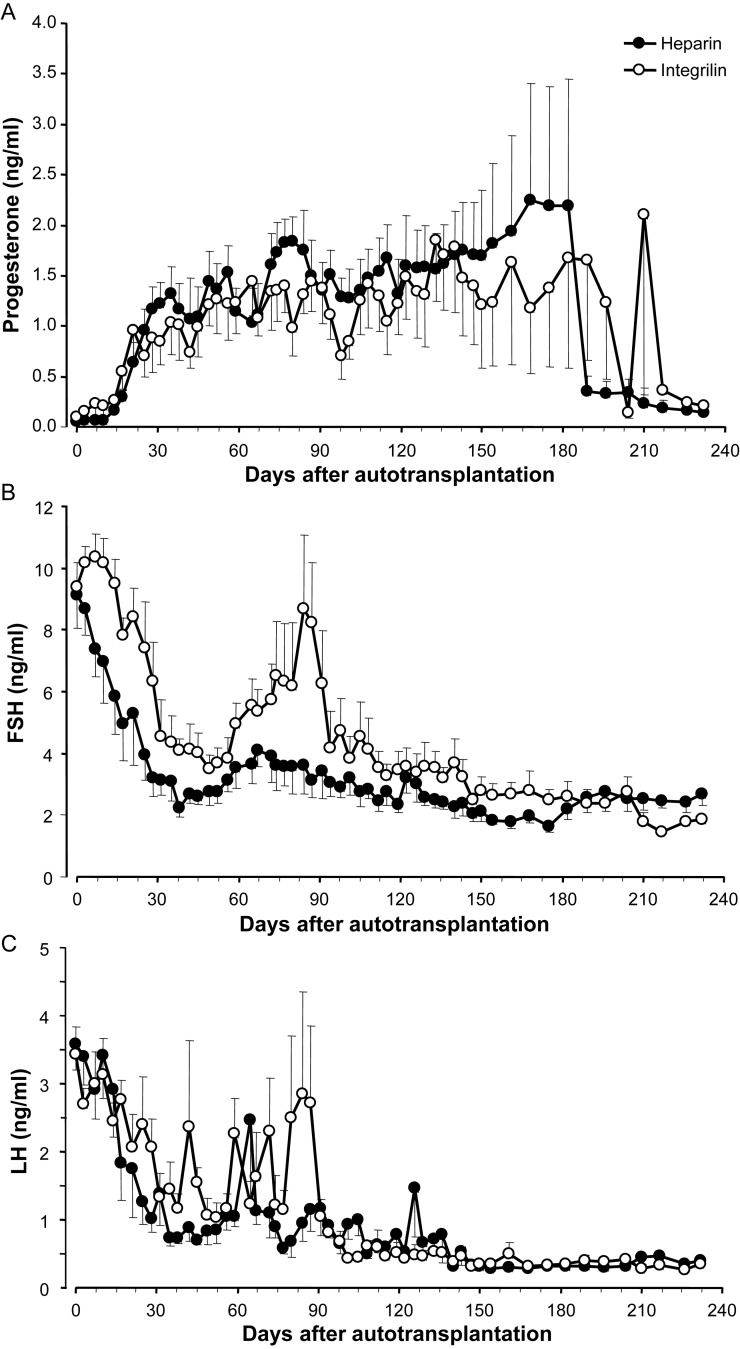
WOCP&TP experiment in 2011–2012 (Study 2); Mean ± SEM progesterone (**A**), FSH (**B**) and LH (**C**) concentrations in jugular venous plasma over the 8 months following autotransplantation of cryopreserved ovaries. Closed symbols illustrate hormone levels in ewes which received post-operative treatment with an anti-coagulant regime consisting of Asp+Hep (LMWH) (*n* = 7) whereas open symbols show data for ewes treated with Asp+Integ (Eptifib) (*n* = 7). From approximately Day 180, data from pregnant animals were no longer included in the profiles so *n* = 5/group for these profiles. Note the rapid restoration of ovarian function as evident from the rapid fall in peripheral FSH and LH concentrations following autotransplantation and rapid increase in progesterone after 14–21 days, indicating ovulation within 2–3 weeks of transplantation. Note also the difference in FSH profiles between anti-coagulant regimes reflecting a higher degree of efficacy for Asp+Hep at this dose.

**Figure 5:**
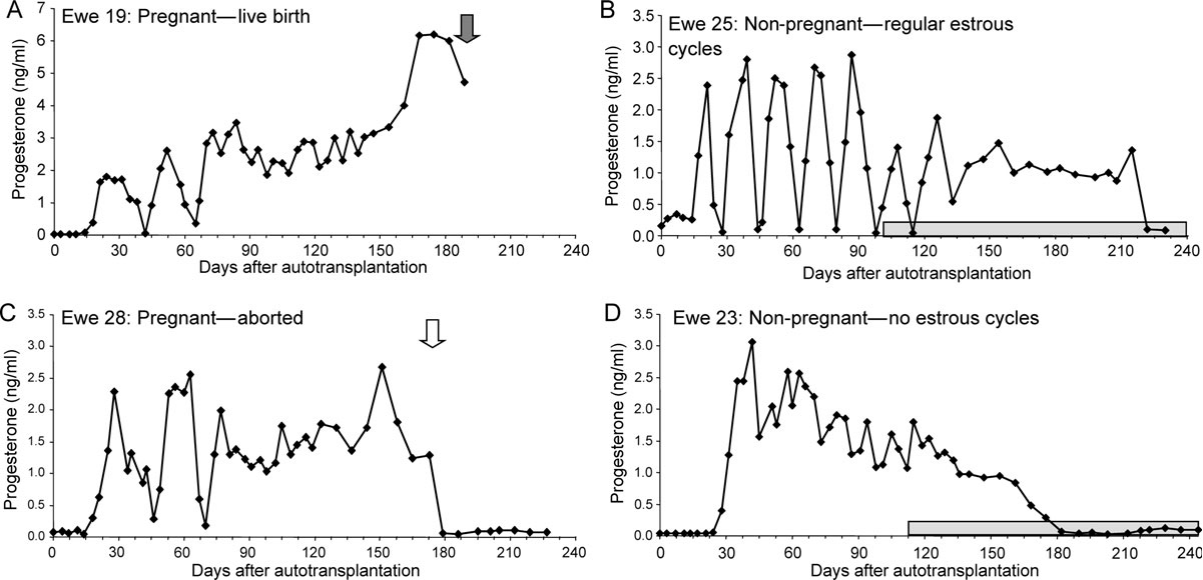
WOCP&TP experiment in 2011–2012 (Study 2); Progesterone profiles from individual ewes illustrating different reproductive outcomes following WOCP&TP. (**A** and **C**) Two animals (Ewes 19 and 28) who became pregnant after showing two spontaneous oestrous cycles with Ewe 19 (A) successfully giving birth to a normal lamb after a normal gestation of ∼140 days and Ewe 28 aborting in the third trimester (Day 106). (**B** and **D**) Two ewes who did not become pregnant. Ewe 25 (B) ovulated within 2 weeks of autotransplantation and had regular oestrous cycles until the beginning of seasonal anoestrus in this species (light grey bar on *x*-axis of figure). It is likely that pregnancy was prevented in this animal due to either damage to the oviducts or mislocation of the ovary. Ewes 23 (D) also ovulated rapidly after autotransplantation but did not cycle with high progesterone being maintained for ∼140 days. Post-mortem confirmed that this animal had a fully functional ovary and it is therefore likely that the surgery had interfered with the normal mechanisms regulating luteal regression in this species.

**Figure 6:**
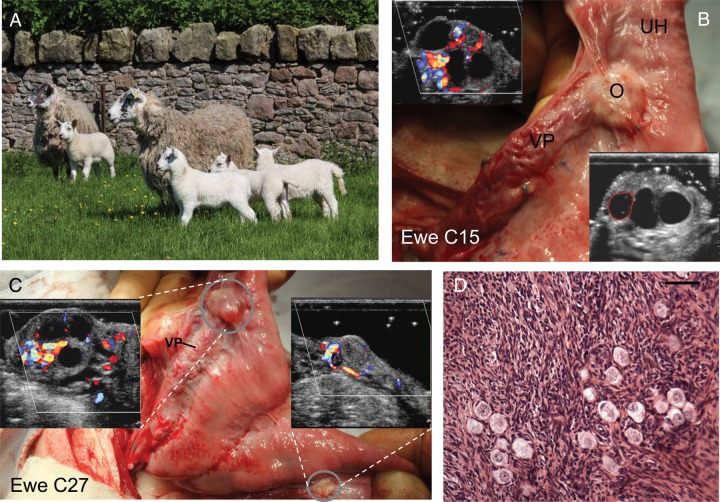
WOCP&TP experiment in 2011–2012 (Study 2). (**A**) Image of 2 of the 4 Greyface ewes that became pregnant following WOCP&TP with their lambs (1 singleton and 1 set of triplets). (**B**) Normal appearance of ovary (O) and vascular pedicle (VP) in a ewe 10 months after WOCP&TP. Right hand insert is conventional ultrasound image showing presence of numerous large antral follicles in the ovary. Left hand inset shows Doppler ultrasound illustrating high levels vascular perfusion. (**C**) Illustration of the effect of different CPA perfusion time on subsequent ovarian function. The left ovary (upper circle) was perfused with CPA for 60 min and right ovary (lower circle) was perfused for 10 min prior to WOCP&TP in same animal. The left ovary appears normal, has well vascularized pedicle (VP) and as shown by left inset has numerous large follicles. The right ovary, despite good post-operative perfusion, was vestigial with few follicles and low blood flow (right inset) 8 months after WOCP&TP. (**D**) Histological image showing a cluster of numerous primordial follicles within an ovary recovered 8 months after WOCP&TP. It is estimated that 70% of primordial follicles survived this procedure and as this represents tens of thousands of follicles, this follicular cohort would enable restoration of ovarian function for several decades. Scale bar 39 µm.

The results of this study were far beyond expectation in terms of the consistency of the response. Apart from two animals removed from the analysis due to procedural errors, all remaining animals (14/14) showed restoration of ovarian function with subsequent ovulation within 2–3 weeks of surgery (Fig. 4A). As further confirmation of the full restoration of ovarian function, over the subsequent 3 month period when the ewes were run with an entire ram, 9/14 (64%) became pregnant (Fig. 5A; Table II) and 4/14 ewes maintained these pregnancies to term, with the birth of a total of seven normal lambs (Figs 5 and 6A; Table II). Subsequent development of these offspring has been normal and one of the females has gone on to establish a normal pregnancy to term with the birth of a normal male lamb. Those ewes that aborted did so mainly during the transition from the second to the third trimester (97 ± 5.1 days, gestation 142–144 days in sheep; Fig. 5C). Those animals that did not get pregnant displayed either regular oestrous cycles (Fig. 5B) until the end of the breeding season or did not cycle and showed persistently high progesterone values for around 90–140 days (Fig. 5D), consistent with maintenance of luteal function due to perturbation of luteal regression mechanisms (Goding *et al.*, 1972), resulting from the anatomical changes made as part of the transplant procedure.

**Table II:** Summary of results from WOCP+TP study conducted in 2011–2012 (Study 2) showing reproductive performance of ewes treated with two different anti-coagulant regimes which consisted of Aspirin and LMWH (Asp+LMWH) or Aspirin and Eptifibatide (Asp−Eptifib).

Characteristic	Asp+LMWH (*n* = 7)	Asp+Eptifib (*n* = 7)	Total/*P*
Proportion ovulated (%) (progesterone >1 ng/ml 14 days)	7/7 (100)	7/7 (100)	14/14 (100)
Proportion showed oestrus (%)	6/7 (86)	6/7 (86)	12/14 (86)
Proportion became pregnant (%)	5/7 (71)	4/7 (57)	9/14 (64)
Proportion lambed (no/sex lambs) (%)	2/7 (3♀, 1♂)	2/7 (1♀, 2♂)	4/14 (29)
AMH (ng/ml)^a^	32.5 ± 7.1	47.2 ± 18.1	1.0
Inhibin A (ng/ml)^a^	0.43 ± 0.06	0.48 ± 0.07	0.20

Of the 16 ewes treated, one from each group was removed from the analysis due to methodological errors during the surgery (see methods for more detail).

^a^Determined 5 months after autotransplantation.

Recovery of ovarian tissue either 8 or 22 months after WOCP&TP from all animals showed clear evidence of a superior outcome in ovaries that had been perfused with CPA for the longer 60 min period. In all instances, the 60 min perfusion resulted in an ovary of normal appearance containing numerous primordial, pre-antral and antral follicles (Table III) with an apparently normal ovarian vascular pedicle with an abundant ovarian blood supply evident on Doppler ultrasound (Fig. 6B–D). In addition, in the four animals that carried live births to term and were sacrificed in the breeding season, all had corpora lutea present in the ovary perfused with CPA for 60 min. In contrast, ovaries perfused with CPA for the shorter 10 min period had either been resorbed completely and were vestigial (6/10; Fig. 6C) or were markedly smaller (*P* = 0.002) than the contralateral ovary in the same animal that had been perfused for 60 min (Table III). These smaller ovaries contained fewer pre-antral (*P* = 0.01) and antral (*P* = 0.02) follicles (Table III) and also had lower levels of blood flow evident from Doppler ultrasound (Fig. 6C) despite the ovarian vascular pedicle appearing to be macroscopically normal and patent. Overall, these results demonstrated clearly that the period of exposure to CPA is a key determinant of the success of the WOCP&TP procedure and that the 60 min perfusion period was superior for the preservation of function in whole adult sheep ovaries.

**Table III:** Summary of results from WOCP+TP study conducted in 2011–2012 (Study 2) showing the effect of CPA perfusion times on the ovarian size and density of ovarian follicles in paired ovaries from each ewe perfused with CPA for either 10 or 60 min prior to cryopreservation.

Characteristic	10 min (*n* = 14)	60 min (*n* = 14)	*P*
Ovarian area section (mm^2^)	113 ± 22	274 ± 25	0.0002
Primordial and pre-antral follicle density (follicles/mm^3^)	1.73 ± 0.27	2.45 ± 0.37	0.013
Antral follicle density (follicles/mm^3^)	0.87 ± 0.08	1.04 ± 0.05	0.021

Follicle density values have been subject to square root (+0.5) transformation prior to analysis and presentation. Data have been pooled across anti-coagulant regimes as there were no significant differences between groups.

Comparison between the efficacy of the two anti-coagulant regimes tested in this experiment revealed no difference in terms of either the proportion of ewes that resumed ovarian function, became pregnant or carried lambs to term (Fig. 4A; Table II). Examination of the effects of the anti-coagulant therapy within different CPA perfusion times showed no significant effect on ovarian size, primordial and pre-antral or antral follicle number (data not shown). Based on the Time 0 primordial and pre-antral follicle density determined from a similar group of animals in Study 1 (12.4 ± 3.8 follicles/mm^3^; non-transformed data), we estimate that ∼60–70% of the primordial follicles survived the WOCP&TP procedure in ovaries perfused with CPA for 60 min, although there was significant between-animal variation. The lack of effect of anti-coagulant on follicle survival was generally consistent with analysis of endocrine markers of ovarian reserve evident between these two treatment groups. As shown in Fig. 4B, all ewes exhibited an acute decline in FSH levels following WOCP&TP which could be attributed to continued growth and development of the growing follicle population. Ewes treated with Asp+LMWH(Hep) exhibited a more acute decline in FSH (13.8 ± 2.2 days) than those treated with Asp+Eptifib(Integ) (27.7 ± 3.1 days; *P* = 0.003) and returned to normal levels within 5–6 weeks of TP. In contrast, ewes treated with Asp+Eptifib(Integ) took 7 weeks to reach a nadir of around 3.5 ng/ml before increasing again to a peak of 8.7 ng/ml at 12 weeks and declining thereafter to normal levels towards the end of the experimental period. As a result of this bimodal pattern of secretion, FSH levels were significantly higher (*P* = 0.03) in Asp+Eptifib treated ewes for 3 months after aTP but did not differ thereafter. As expected, mean LH values exhibited a similar decline following TP as was observed with FSH but overall LH values did not differ significantly with anti-coagulant treatment (Fig. 4C). As expected, both AMH and inhibin A levels increased significantly (*P* < 0.01) over the initial 3–4 months after aTP and then remained relatively stable thereafter (data not shown). There were no significant differences in the patterns of AMH or inhibin A levels post-transplantation in ewes which received the different anti-thrombotic regimes (Table II).

## Discussion

Although a single live birth has previously been reported from one of nine lambs that had undergone WOCP&TP (Imhof *et al.*, 2006), this paper is the first to report high rates of acute restoration of ovarian function and fertility in large animals with multiple live births following cryopreservation and aTP of whole adult ovaries. As far as we are aware this work also represents the first report of full restoration of functionality following freezing of any adult organ from a large mammal.

This breakthrough has been achieved through optimization of freezing and thawing protocols which maximize cryoprotection whilst minimizing cytotoxicity during cryopreservation and by utilizing anti-thrombotic agents post-operatively to prevent post-operative ischaemia in the cryopreserved organ due to clot formation in the organ vasculature resulting from endothelial cell cryodamage and cytotoxicity. These innovations provide valuable insights into how whole organ preservation and transplantation could be used to preserve and restore fertility in girls and women at risk of POF. They also provide proof of principle for potential applications involving the cryopreservation of other human organs.

The successful application of WOCP&TP to mature adult animals in these studies reinforces the potential advantages of WOCP&TP over the alternate intervention of ovarian cortex autografting. By preserving the entire ovarian reserve within the whole ovary and minimizing primordial follicle loss due to post-operative ischaemia by acute restoration of blood flow to the transplant by vascular anastomosis, we have achieved acute restoration of ovarian function and fertility without significant endocrine disturbance and importantly have incurred very low rates of primordial follicle loss compared with the levels incurred during cortex autografting (Baird *et al.*, 1999). The surviving follicle population of ∼30–40 000, estimated in ovaries perfused with CPA for 60 min from animals treated with aspirin+LMWH, would be expected to be sufficient to restore ovarian function and fertility in a human context for many decades (Wallace and Kelsey, 2010). Indeed higher rates of follicle survival (>70%) should be achievable with further procedural optimization. While it is true that the period of restoration of fertility following autografting of ovarian cortex in humans (around 5 years; Silber, 2012; Donnez *et al.*, 2013) has been longer than that demonstrated in earlier sheep studies (2 years; Baird *et al.*, 1999), it is clear that cortex autografting is most effective in younger women with higher ovarian reserves (Donnez *et al.*, 2013). Given the greater surgical complexity, the all-or-none nature of the intervention and the greater risk of re-introduction of malignant tissue in cancer survivors, WOCP&TP, when compared with cortex autografting, may therefore prove most valuable as an alternative treatment in older women where autografting of fragments of the cortex is unlikely to be as successful or conversely in young girls where the ability to restore full reproductive longevity would be advantageous. However, in order to make these options a reality, further optimization with animal experimentation is required (see below).

The importance of optimizing exposure time to CPA in order to allow adequate penetration of CPA, whilst minimizing CPA toxicity, was clearly illustrated in this study by the difference in ovarian function observed between ovaries infused with CPA for either 10 or 60 min prior to WOCP. Despite *in vitro* results indicating less tissue damage in ovaries perfused for the shorter time period (Fig. 4B), the longer time period was markedly superior following aTP *in vivo*, presumably because of the much higher degree of CPA penetration achieved to the ovarian cortex after 60 min of perfusion (Fig. 4A). The extremely rapid restoration of ovarian function achieved following WOCP&TP in some ewes in Study 1 and in all ewes in Study 2 (2–3 weeks) is consistent with results from follicle dissection and culture experiments showing that the 60 min WOCP protocol does not adversely affect the viability of gonadotrophin-responsive follicles up to the small antral stage (1–2.3 mm in diameter; V Onions and BK Campbell, unpublished observations). Although we can conclude that this period of CPA exposure is optimal for WOCP of adult sheep ovaries, the application of this technology to girls and adult women, in which the ovaries are smaller and larger respectively, would require further optimization utilizing other animal model ovaries of an appropriate size (e.g. lambs, cows) and/or donated human ovaries. Despite there being a number of reports of high rates of post-thaw tissue viability in a small number of donated human ovaries processed for WOCP in the literature (Bedaiwy *et al.*, 2006; Jadoul *et al.*, 2007; Martinez-Madrid and Donnez, 2007), in our experience acute *in vitro* estimates of post-thaw viability are poor predictors of long-term ovarian function *in vivo* following WOCP&TP (Onions *et al.*, 2008, 2009). Thus the results of the present studies emphasizes the need to fully optimize the cryopreservation protocol for each type of ovary before attempting aTP.

The other major intervention which was critical to the success of the WOCP&TP procedure was the pre- and post-operative use of anti-thrombotics to prevent acute clot formation in the frozen-thawed vasculature of the cryopreserved organ. The rationale for their use was based on our previous observations that (i) high rates of follicle loss were often observed following WOCP&TP despite long-term maintenance of ovarian vascular supply (Onions *et al.*, 2008, 2009) and that (ii) clot formation in the ovarian vasculature occurred acutely following WOCP&TP and this was associated with up-regulation of endothelial gene expression of factors associated with vascular tone and apoptosis (Onions *et al.*, 2013). The critical nature of this intervention to the success of the procedure is clearly illustrated by comparing the results of Study 1 (2010–2011) WOCP&TP experiment with that of Study 2 (2011–2012). Both these experiments utilized the 60 min perfusion period, but in 2010–2011, where ewes were treated with aspirin alone, only 4/15 (27%) displayed restoration of ovarian function and in those animals follicle survival was low (20%). In contrast, in Study 2 (2011–2012) where animals were treated with a combination of aspirin and enoxaparine (Clexane^®^) or eptifibatide (Integrilin^®^), all animals showed restoration of ovarian function and follicle survival was much higher (60–70%). The use of an optimized anti-thrombotic regime is therefore critical to the success of this procedure. Of the two anti-thrombotics tested in 2011–2012, although there was no difference between the regimes in terms of the rate of restoration of ovarian function and fertility, the Asp+LMWH regime appeared marginally superior to the Asp+Eptifib in terms of follicle survival, as evidenced by both direct ovarian counts (Table II) and differences in FSH levels (Fig. 4). However, this result needs to be treated with some caution as the doses of both these drugs require optimization for this application. Further experimentation is therefore required to determine whether more aggressive anti-thrombotic regimes, involving longer periods of post-operative treatment and/or higher doses, would have a further beneficial effect on primordial follicle survival following WOCP&TP.

Although these studies indicate that WOCP&TP may be a viable clinical intervention for restoration of fertility in women, there are a number of concerns that need to be addressed before the clinical application of this technology. The first of these concerns is the fact that although all animals had functioning ovaries, a number of the animals did not show regular oestrous cycles and/or did not get pregnant despite displaying regular cycles. Both of these effects can be attributed to the degree of the surgical intervention required to perform the double ovarian autotransplant. Luteal regression in sheep, unlike the human, is regulated by the release of prostaglandin F2α (PGF2α) from the non-pregnant uterus which passes by a counter-current mechanism from the utero-ovarian vein to the ovarian artery (McCracken *et al.*, 1972). In Study 2, it was necessary to anastomose the ovarian artery directly to the uterine artery and it is therefore likely that this surgical intervention resulted in perturbation of this luteolytic signal transfer mechanism and hence long-term maintenance of the corpus luteum that resulted from the first ovulation after aTP in some animals (Fig. 5D). Similarly, in animals which cycled regularly but did not get pregnant (Fig. 5B), it is likely that the positioning of the transplanted ovary and/or post-operative adhesions had prevented oocyte pick-up by the oviduct and this was confirmed at the time of post-mortem. In a clinical context, these complications could therefore be minimized by a less radical surgical intervention in which normally only one frozen ovary would be autotransplanted to an optimal site for both reanastomosis of the ovarian vasculature and maintenance of oviduct function. Further, it is well established that, unlike the luteolytic mechanism controlling maintenance of the corpus luteum in sheep, a luteotrophic mechanism mediated by the release of hCG from the developing conceptus operates in primates (Bazer *et al.*, 2010). It therefore appears unlikely that either of these complications would have a major impact in the clinical application of this intervention although importantly, fertility restoration in women following WOCP&TP need not be totally reliant on the success of natural ovulation as it can be supported through the use of established assisted reproduction interventions.

In conclusion, the results of this study demonstrate that full restoration of ovarian function and high rates of fertility can be obtained following WOCP&TP by optimization of CPA penetration during whole ovary perfusion along with the use of anti-thrombotic agents to prevent post-operative clot formation in the ovarian vasculature. It is hoped that this work represents a major advance toward the possible development and clinical application of WOCP&TP as a means to preserve and restore the fertility of girls and women at risk of POF. However, before WOCP&TP can be used clinically, further animal model studies are required to (i) develop optimized cryopreservation protocols for a range of different sized ovaries similar to those observed in prepubertal girls, adolescents and mature women and (ii) generate more information on the optimal type, dose and duration of post-operative anti-thrombotic therapy.

## Authors' roles

B.K.C.: study conception and design; surgery; manuscript preparation; J.H.-M.: cryopreservation and surgical assistance (Study 1 and 2); V.O.: cryopreservation and surgical assistance (Study 1); C.P.-A.: immunoassays; F.A.: LDH determinations; J.F.: NMR analysis; A.S.M.: high resolution ultrasonography and Doppler analysis; R.W.: study conception and design; manuscript preparation; H.M.P.: study conception and design; manuscript preparation.

## Funding

Funding for this study was received from the Medical Research Council, University of Nottingham. Funding to pay the Open Access publication charges for this article was provided by the MRC and University of Nottingham.

## Conflict of interest

None declared.
